# Effect of nitrogen regulation on berry quality and flavonoids during veraison stage

**DOI:** 10.1002/fsn3.2503

**Published:** 2021-08-13

**Authors:** Yueran Hui, Jing Wang, Tingting Jiang, Tinghui Ma, Rui Wang

**Affiliations:** ^1^ College of Agronomy Ningxia University Yinchuan China; ^2^ Ningxia Academy of Agriculture and Forestry Sciences Yinchuan China; ^3^ Ningxia Grape and Wine Research Institute Yinchuan China; ^4^ China Wine Industry Technology Institute Yinchuan China

**Keywords:** anthocyanins, flavanols, flavonols, nitrogen fertilizer, wine grape quality

## Abstract

Nitrogen regulation can effectively promote the improvement of berry components and the formation of flavor compounds in wine grapes. In order to understand the effects of foliar nitrogen spraying on grape quality and flavonoid substance, took Cabernet Sauvignon as the test subject, grape leaves were sprayed by ammonium sulfate, calcium ammonium nitrate, urea, phenylalanine, and glutamate during veraison, and clear water was used as the control. The results showed that spraying ammonium sulfate could improve the contents of soluble solids, anthocyanins, and total phenols of grape berries; spraying phenylalanine significantly increased the content of titratable acid and tannin and decreased the ratio of sugar to acid in grape berries; compared with the control group, spraying glutamate could significantly upregulate some flavonol monomers; spraying calcium ammonium nitrate can adjust the monomer content of some flavanols; urea spraying significantly increased the contents of most anthocyanins, flavanols, and flavonol and increased the contents of total anthocyanins, total flavanols, and total flavonol in grape skins, laying a foundation for the improvement of the nutritional value of grapes and wine in the future.

## Graphical abstract

In this experiment, ammonium sulfate, calcium ammonium nitrate, urea, phenylalanine and glutamic acid were sprayed on the leaves during veraison stage period of the grapes，spraying clean water on the leaves as a control. The results showed that spraying urea had a significant effect on the up‐regulation of most anthocyanins, flavanols and flavonol monomers, and increased the content of total anthocyanins, total flavanols, and total flavonols in grape skins.

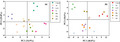

## 1 INTRODUCTION

Nitrogen is an essential nutrient element in plant growth and development (Zhao et  al., 2017), and rational utilization of nitrogen affects the absorption of mineral nutrients, photosynthate, and hormone levels of plants, and further affects the growth and development of fruit trees and fruit yield (Chen et  al., 2018; Miquel et  al., 2016; Zhao et  al., 2018). Appropriate application of nitrogen fertilizer could significantly increase leaf nitrogen content, dry matter weight, and yield (Zhang et  al., 2007), and excessive application of nitrogen fertilizer will lead to problems such as overgrowth of tree body nutrition, decreased fruit setting rate, and yield (Zhang et  al., 2017). Restricting nitrogen application in vineyards can prevent excessive vegetative growth of grape and improve grape quality (Bell & Henschke, 2005). However, excessive nitrogen control will lead to the decrease in nitrogen content in grapes, and the failure to meet the normal nitrogen metabolism requirements of yeast in the process of alcohol fermentation, leading to fermentation stop (Bell & Henschke, 2005; Garde‐Cerdán & Ancín‐Azpilicueta, 2008; Gutiérrez‐Gamboa et  al., 2017). Leaf nitrogen spraying did not affect the nutritional growth of wine grapes, but could promote the nitrogen content in the fruit, and provide sufficient yeast assimilable nitrogen for the later grape alcohol fermentation (Gutiérrez‐Gamboa et  al., 2017; Hannam et  al., 2016).

Phenols are one of the most important secondary metabolites in grape (Cheng et  al., 2020), including flavonoids and nonflavonoids; flavonoids can be divided into anthocyanins, flavanols, and flavonols, while nonflavanoids can be divided into resveratrol, cinnamic acid, and so on (Liang et  al., 2013). Flavonoids are the most abundant phenolic substances in grapes and wine, which are mainly distributed in the pericarp and seed coat. They can resist the damage of ultraviolet radiation and pathogens to grapes and have antioxidant functions (Bin et  al., 2019), and it also plays an important role in the quality parameters of red wine (Gonzalo et  al., 2019; Ma et  al., 2020). These compounds are produced by the flavonoid metabolic pathway (Wei et  al., 2020); the most important precursor of this pathway is phenylalanine (Javier et  al., 2017); moreover, spraying phenylalanine on the leaves could promote the synthesis of phenolic compounds in grapes (Javier et  al., 2017). Flavonoids synthesis in grapefruits was most active in the late stage of color turning. Flavonoids in grape and wine could be effectively improved by rapid nitrogen supplementation on leaf surface (Cheng et  al., 2020). Carina et  al., (2019) found that nitrogen fertilizer could significantly affect the aroma and sensory characteristics of grapes and wine, and the levels of 33 metabolites in leaves and 55 metabolites in wine were significantly different due to the application of fertilizers with different nitrogen forms. Javier et  al., (2015) found in the study of the effects of foliar spraying of phenylalanine and urea on grape flavonoid substances that 0.9  kg  N/ha urea could increase the monomer content of several anthocyanins and flavonols. Some studies have also shown that the application of nitrogen fertilizer before the turning period has no significant effect on the flavonoids and volatile compounds in grape wine and wine due to the influence of special environmental factors, such as water shortage and sunlight (Garde‐Cerdán et  al., 2015; Javier et  al., 2017; Martínez‐Lüscher et  al., 2017).

There are many studies on the effects of soil nitrogen application and nitrogen application amount on the fermentation, quality, and yeast assimilated nitrogen of wine. However, the effects of nitrogen regulation at the veraison stage on grape quality and flavonoid substances are less studied. The aim of this study was to understand the effects of nitrogen regulation on grape berry composition and flavonoid compounds in *Cabernet Sauvignon* vineyards during the color turning period, so as to provide support for grape quality improvement and wine fermentation.

## 2 MATERIALS AND METHODS

### 2.1 Test design

The experimental site was located in Lilan Chateau (105°58′20″ E, 38°16′38″ N) in Yongning County, Ningxia. The soil type is gravelly light lime soil, and the soil texture was gravelly sand soil. The experimental grapes were 8 years old *Cabernet Sauvignon*, the planting direction was north‐south, the tree shape was “Inclined upper frame shape,” the plant row spacing was 0.6 m × 3.5 m, and the irrigation method was drip irrigation.

There were six treatments in the experiment, which were spraying ammonium sulfate (AS), calcium ammonium nitrate (CAN), urea (Ur), phenylalanine (Phe), glutamate (Glu), and clear water (control), and the amount of nitrogen fertilizer in each treatment was converted to 1.5‰ (Table 1). A single‐factor random block design was adopted in the experiment. Each treatment had 5 replicates, and there were 30 plots in total, with a total area of 1,890  m^2^. Spraying was carried out three times (July 15, July 31, and August 13) during the grape veraison stage, and irrigation, pruning, pest control, and other production management measures were consistent.

**TABLE 1:** Types and dosages of nitrogen sources

Nitrogen source type	*N* content (%)	Nitrogen application rate (g/m^2^)
Control	‐	0
Ammonium sulfate (AS)	21.2	0.26
Calcium Ammonium nitrate (GAN)	23.0	0.24
Urea (Ur)	46.7	0.12
Phenylalanine (Phe)	8.5	0.64
Glutamate (Glu)	9.5	0.58

### 2.2 Determination of grapefruit quality

Soluble solids were measured with a handheld sugar meter, and the titratable acid content was determined by the standard 0.1  mol·L^‐1^ NaOH method (Jin et  al., 2016); reducing sugar was determined by anthrone reagent method (Sohrab et  al., 2016); the total phenol was determined by Foling–Shocka method. Tannins were determined by Flynn–Dennis method, and anthocyanins were determined by the pH differential method (Yang, 2016).

### 2.3 Analysis of flavonoids in grape

#### 2.3.1 Preparation and extraction of flavonoids from grapes

The sample was placed in a freeze‐drying machine (SCIENTZ‐100F) for vacuum freeze‐drying, and the grinding machine (MM400, Retsch) was used to grind the sample at 30 Hz for 1.5 min to powder form. 100 mg of the freeze‐dried powder was dissolved in 1.2 ml 70% methanol solution for vortex and then placed in a refrigerator at 4℃ after vortex. The sample was centrifuged at 12,000 rpm for 10 min and then filtered with a microporous membrane (pore size was 0.22 μm) for later use.

#### 2.3.2 Chromatographic analysis of flavonoids in grapes

Sample extracts were analyzed by UPLC‐ESI‐MS/MS system (UPLC, SHIMADZU Nexera X2, www.shimadzu.com.cn /; MS, Applied Biosystems 4,500 Q TRAP, www.appliedbiosystems.com.cn /). The analysis conditions are as follows: UPLC: chromatographic column, Agilent SB‐C18 (1.8 µm, 2.1 mm * 100 mm); the mobile phase consists of solvent A: pure water containing 0.1% formic acid and solvent; B: acetonitrile containing 0.1% formic acid. Use gradient program to measure samples, which uses the initial conditions of 95% A and 5% B; within 9 min, the proportion of phase B increased linearly to 95% and remained at 95% for 1 min. In 10–11.1 min, the proportion of phase B was reduced to 5%, and the balance was 5% to 14 min. The flow rate was set at 0.35 ml/min. The temperature of the column box is set to 40℃; the amount of injection was 4 μl.

The LIT and triple quadrupole (QQQ) scans were obtained on triple quadrupole linear ion trap mass spectrometer (Q TRAP), AB4500 Q TRAP UPLC/MS/MS system, and the system is equipped with ESI Turbo Ion‐Spray in 29 interface, which has two negative ion modes, and is controlled by Analyst 1.6.3 software (AB SCIEX). The instrument was tuned and calibrated with 10 and 100 μmol/L polypropylene glycol solutions in QQQ and LIT modes, respectively. QQQ scan was obtained through MRM experiment, and the collision gas (nitrogen) was set to medium. Through further DP and CE optimization, DP and CE for a single MRM transition were completed. A specific set of MRM ion pairs was monitored in each period based on the metabolites eluted in each period.

### 2.4 Statistical analysis

Microsoft Excel 2016 and SPSS 24.0 were used to process and analyze the data, and origin 2018 was used to plot the data, and the significant level was (*p* < .05, *n* = 5).

## 3 RESULTS AND ANALYSIS

### 3.1 Effects of nitrogen regulation on grape and berry components during veraison stage

AS treatment significantly improved the contents of TSS, TA, and TP in grape berries, while Phe treatment increased the contents of TAC and TN in grape berries and decreased the contents of TSS/TAC (Table 2). AS treatment significantly increased the content of TSS; the TAC content of grape berries was the highest under Phe treatment, followed by GAN >Ur> AS. The TSS/TAC of grape berries under AS treatment was significantly higher than that of control and other treatments. Compared with control, AS, GAN, and Phe treatments significantly increased TN content in grape berries, Phe treatment was the highest, next is AS and GAN treatment, and they increased by 26.84%, 18.45%, and 15.78% in turn. AS and Ur treatments significantly increased the content of TA in grape berries, and they are increased by 79.05% and 78.37%, respectively. AS treatment also significantly increased the TP content of grape berries.

**TABLE 2:** Effects of nitrogen regulation on the composition of Cabernet Sauvignon berries in the veraison stage

Indexes	TSS	TAC	TSS/TAC	RS	TN	TA	TP
Control	24.53 ± 0.18c	0.58 ± 0.01c	42.08 ± 0.88e	181.16 ± 3.89a	15.72 ± 0.53b	1.48 ± 0.01b	14.89 ± 0.17c
AS	29.07 ± 0.34a	0.61 ± 0.01bc	47.91 ± 0.46a	173.01 ± 21.14a	18.62 ± 0.80a	2.65 ± 0.02a	21.13 ± 0.41a
GAN	28.63 ± 0.33ab	0.67 ± 0.03b	43.21 ± 2.66d	160.62 ± 11.85a	18.20 ± 0.68a	2.07 ± 0.46ab	15.43 ± 0.20c
Ur	28.67 ± 0.18ab	0.65 ± 0.02b	43.97 ± 1.54c	173.82 ± 14.25a	15.30 ± 0.55b	2.64 ± 0.19a	14.84 ± 0.09c
Phe	26.93 ± 1.27b	0.85 ± 0.05a	31.92 ± 3.31f	189.23 ± 7.70a	19.94 ± 0.52a	1.46 ± 0.34b	17.83 ± 0.57b
Glu	24.67 ± 0.24c	0.56 ± 0.05c	44.78 ± 4.08b	167.28 ± 7.88a	15.46 ± 0.33b	1.98 ± 0.20ab	15.24 ± 0.31c

Note

All the parameters are given with their standard deviation (*n* = 5). TSS = total soluble solids (%). TAC: titratable acid content (expressed in gram equivalent tartaric acid L^−1^). RS: reducing sugar (expressed in gram equivalent glucose L^−1^). TN, tannins (mg tannin/100 g fresh weight); TA, anthocyanins (mg anthocyanin/100 g fresh weight); TP, total phenols (mg gallic acid/100 g fresh weight); Different lowercase letters indicate significant differences between treatments as calculated by Tukey's HSD test (*p* < .05).

### 3.2 Effects of nitrogen regulation on anthocyanins in grape skins during veraison stage

GAN treatment significantly improved Pt and cMvcoum, Phe treatment significantly improved Mvacet, and Ur treatment significantly improved the content of total anthocyanins in grape skins (Table 3). There were 22 anthocyanins in grape skins by UPLC‐MS analysis, and they are mallow malvidin class (5), cyanidin class (5), peonidin class (4), petunidin class (4), delphinidin class (3), and pelargonidin class (1). From the perspective of individual anthocyanins, the contents of Mv and its derivatives were increased by nitrogen regulation at the veraison stage. The contents of Pt and cMvcoum in grape skins under GAN treatment were significantly higher than those under other treatments. Under Phe treatment, the content of Mvacet in grape peel was the highest, which increased by 18.35% compared with control; except for Cy, Pngluc, Pt, and cMvcoum, almost all single anthocyanins in Ur treatment were significantly higher than those in other treatments; In terms of total anthocyanins, the content of total anthocyanin in grape skins under Ur treatment was the highest, which increased by 12.73%, 28.56%, 23.21%, 17.18%, and 16.50%, respectively, compared with control, AS, GAN, Phe, and Glu.

**TABLE 3:** Effects of nitrogen regulation on anthocyanins in grape skins during veraison stage

Flavonoids	Control	AS	GAN	Ur	Phe	Glu
Cy	1.16 ± 0.12a	0.52 ± 0.13b	0.67 ± 0.07b	0.70 ± 0.12b	0.56 ± 0.05b	0.46 ± 0.02b
Pl	1.05 ± 0.05a	0.73 ± 0.07b	0.68 ± 0.01b	0.93 ± 0.06a	0.93 ± 0.06a	0.92 ± 0.03a
Pn	1.12 ± 0.07cd	0.98 ± 0.03e	1.01 ± 0.01de	1.45 ± 0.04a	1.23 ± 0.04bc	1.32 ± 0.04b
Cygala	0.89 ± 0.09b	0.54 ± 0.04d	0.58 ± 0.02d	1.06 ± 0.03a	0.68 ± 0.02cd	0.79 ± 0.05bc
Cygluc	1.04 ± 0.02a	0.51 ± 0.06c	0.60 ± 0.02bc	0.94 ± 0.09a	0.67 ± 0.02b	0.74 ± 0.03b
Pngluc	1.01 ± 0.02a	0.72 ± 0.05d	0.76 ± 0.04cd	0.95 ± 0.04ab	0.87 ± 0.04bc	0.90 ± 0.05ab
Mv	0.98 ± 0.02c	1.07 ± 0.03bc	1.21 ± 0.01bc	1.46 ± 0.04a	1.41 ± 0.01a	1.39 ± 0.1a
Pt	0.98 ± 0.01a	0.77 ± 0.04b	1.02 ± 0.02a	0.82 ± 0.04b	0.83 ± 0.03b	0.83 ± 0.05b
Cyacet	1.08 ± 0.04a	0.64 ± 0.03b	0.68 ± 0.01b	1.01 ± 0.1a	0.71 ± 0.02b	0.69 ± 0.04b
Pncaff	0.85 ± 0.04abc	0.79 ± 0.06abc	0.74 ± 0.02bc	0.89 ± 0.06a	0.87 ± 0.02ab	0.71 ± 0.05c
Dpacet	1.01 ± 0.04a	0.64 ± 0.04b	0.74 ± 0.04b	1.01 ± 0.12a	0.90 ± 0.02ab	0.76 ± 0.14ab
Ptacet	0.98 ± 0.02a	0.83 ± 0.02a	0.97 ± 0.02a	1.00 ± 0.04a	0.88 ± 0.02a	0.84 ± 0.11a
Mvacet	0.89 ± 0.06b	0.97 ± 0.02ab	1.08 ± 0.04ab	1.08 ± 0.02ab	1.09 ± 0.05a	1.02 ± 0.1ab
Mvmalo	0.88 ± 0.06b	0.93 ± 0.02b	1.10 ± 0.03ab	1.29 ± 0.06a	1.31 ± 0.05a	1.20 ± 0.15a
Cycoum	1.10 ± 0.05a	0.84 ± 0.01c	1.00 ± 0.03ab	1.14 ± 0.05a	0.76 ± 0.03c	0.85 ± 0.09bc
Decoum	1.03 ± 0.06ab	0.85 ± 0.11b	0.64 ± 0.05c	1.07 ± 0.01a	0.62 ± 0.03c	0.87 ± 0.08ab
Ptarab	1.11 ± 0.15b	0.73 ± 0.11c	1.21 ± 0.12b	1.81 ± 0.05a	1.14 ± 0.1b	1.31 ± 0.16b
Ptcoum	0.96 ± 0.04b	0.87 ± 0.01c	0.71 ± 0.04d	1.42 ± 0.00a	0.82 ± 0.01c	0.97 ± 0.01b
Pndigl	1.08 ± 0.04b	0.94 ± 0.09b	0.72 ± 0.04c	1.32 ± 0.03a	0.94 ± 0.04b	0.97 ± 0.08b
Dp	0.98 ± 0.01bc	1.14 ± 0.08ab	0.76 ± 0.06c	1.25 ± 0.12a	1.04 ± 0.09ab	0.98 ± 0.08bc
cMvcoum	1.03 ± 0.03bc	0.93 ± 0.02c	1.63 ± 0.05a	0.93 ± 0.04c	1.06 ± 0.03b	1.12 ± 0.06b
Mvdi	0.79 ± 0.04d	1.06 ± 0.13bcd	0.85 ± 0.09cd	1.68 ± 0.28a	1.56 ± 0.16ab	1.35 ± 0.15abc
Total anthocyanins	22.00 ± 1.58b	18.00 ± 0.82d	19.36 ± 0.19c	25.21 ± 0.60a	20.88 ± 0.53b	21.04 ± 1.22b

Note

Different lowercase letters indicate significant differences between treatments as calculated by Tukey's HSD test (*p* < .05).

Abbreviations: Cy: Cyanidin; Dp: Pelargonidin‐3‐*O*‐glucoside; Pl: Peonidin‐3‐*O*‐arabinoside; Cygala: Cyanidin‐3‐*O*‐galactoside*; Cygluc: Cyanidin‐3‐*O*‐glucoside (Kuromanin)*; Pngluc: Peonidin‐3‐*O*‐glucoside; Mv: Malvidin‐3‐*O*‐arabinoside; Pt: Petunidin‐3‐*O*‐glucoside; Cyacet: Cyanidin‐3*‐O*‐(6''‐*O*‐acetyl) glucoside; Pncaff: Peonidin‐3‐*O*‐(6''‐*O*‐Acetyl) glucoside; Dpacet: Delphinidin‐3‐*O*‐(6''‐*O*‐acetyl) glucoside; Ptacet: Petunidin‐3‐*O*‐(6''‐*O*‐Acetyl) glucoside; Mvacet: Malvidin‐3‐*O*‐(6''‐*O*‐acetyl) glucoside; Mvmalo: Malvidin‐3‐*O*‐(6''‐*O*‐malonyl) glucoside; Cycoum: Cyanidin‐3‐*O*‐(6''‐*O*‐p‐Coumaroyl)glucoside; Decoum:Delphinidin‐3‐*O*‐(6''‐*O*‐p‐coumaroyl) glucoside; Ptarab: Petunidin‐3‐*O*‐glucoside‐5‐*O*‐arabinoside; Ptcoum: Petunidin‐3‐*O*‐(6''‐*O*‐p‐Coumaroyl) glucoside; Pndigl: Peonidin‐3,5‐*O*‐diglucoside; Dp: Delphinidin‐3,5‐di‐*O*‐glucoside; cMvcoum: Malvidin‐3‐*O*‐(6''‐*O*‐p‐coumaroyl) glucoside; Mvdi: Malvidin‐3,5‐di‐*O*‐glucoside (Malvin).

### 3.3 Effects of nitrogen regulation on flavanols and flavonols in grape skins during veraison stage

The main flavanols in grape skins are catechin, followed by epicatechin. Among the 19 flavanols, catechin and its derivatives and epicatechin and its derivatives account for 63.16% of the total (Table 4). From the perspective of individual flavanols, the contents of Meepi, Epgal, and Cagal could be increased by nitrogen regulation during the chromaticity stage. Under GAN treatment, the contents of Hydro, Ga, Epgal, Cagal, βCadih, and Epiga were significantly higher than those of other treatments. Except for Hydro, Epiga, Na, and Gagal, the content of almost all flavanols in grape skin treated by Ur was higher than that of control; under Ur treatment, the content of total flavanols in grape pericarp was the highest, which was 16.30%, 52.19%, 26.87%, 32.93%, and 27.00% higher than that of control, as, GAN, Phe, and Glu, respectively.

**TABLE 4:** Effects of nitrogen regulation on flavanols and flavonols in grape skins during veraison stage

Flavonoids	Control	AS	GAN	Ur	Phe	Glu
Ca	0.94 ± 0.03b	0.28 ± 0.06d	0.68 ± 0.02c	1.08 ± 0.01a	0.66 ± 0.03c	0.68 ± 0.02c
Pe	0.96 ± 0.04bc	0.31 ± 0.06d	1.15 ± 0.04ab	1.20 ± 0.11a	0.81 ± 0.04c	0.84 ± 0.06c
Ep	1.05 ± 0.03b	0.38 ± 0.1d	1.16 ± 0.04ab	1.26 ± 0.07a	0.81 ± 0.03c	0.99 ± 0.06bc
Hydro	1.07 ± 0.04a	0.99 ± 0.04a	1.09 ± 0.04a	0.98 ± 0.04a	1.01 ± 0.03a	1.03 ± 0.06a
Medic	3.16 ± 2.13a	1.02 ± 0.16a	0.63 ± 0.26a	2.45 ± 1.27a	1.49 ± 0.65a	0.86 ± 0.13a
Meepi	0.77 ± 0.40b	0.94 ± 0.08b	0.96 ± 0.19b	1.07 ± 0.06b	1.64 ± 0.4b	2.69 ± 0.28a
Ga	0.95 ± 0.03b	0.46 ± 0.10c	1.15 ± 0.01a	1.12 ± 0.03a	0.92 ± 0.01b	0.86 ± 0.02b
Epcat	0.98 ± 0.04a	0.40 ± 0.08c	1.01 ± 0.02a	1.03 ± 0.04a	0.81 ± 0.01b	0.83 ± 0.02b
Epgal	0.93 ± 0.04b	0.94 ± 0.09b	1.70 ± 0.09a	1.47 ± 0.4ab	1.20 ± 0.14ab	1.57 ± 0.11a
Cagal	0.99 ± 0.02b	1.00 ± 0.18b	2.16 ± 0.15a	1.73 ± 0.09a	1.83 ± 0.18a	1.74 ± 0.13a
Cid	0.90 ± 0.08a	0.45 ± 0.02c	0.86 ± 0.04a	0.94 ± 0.03a	0.63 ± 0.02b	0.57 ± 0.06bc
βCadih	0.95 ± 0.05b	0.96 ± 0.12b	1.39 ± 0.05a	1.23 ± 0.08a	0.80 ± 0.03b	0.84 ± 0.06b
αCadih	0.96 ± 0.03a	0.48 ± 0.04d	0.70 ± 0.01b	0.97 ± 0.03a	0.56 ± 0.03cd	0.60 ± 0.04bc
Cic	0.99 ± 0.01b	0.45 ± 0.08d	0.98 ± 0.06b	1.23 ± 0.03a	0.64 ± 0.04c	0.60 ± 0.04c
Epglu	1.06 ± 0.06b	0.66 ± 0.14c	0.69 ± 0.04c	1.36 ± 0.03a	0.72 ± 0.03c	0.66 ± 0.08c
Epiga	1.06 ± 0.04b	0.63 ± 0.07c	1.34 ± 0.09a	1.04 ± 0.13b	0.96 ± 0.06b	0.89 ± 0.09b
Na	0.97 ± 0.03a	0.58 ± 0.02c	0.56 ± 0.01c	0.79 ± 0.06b	0.72 ± 0.02b	0.93 ± 0.01a
Gagal	1.39 ± 0.45a	1.18 ± 0.33a	0.53 ± 0.05a	1.40 ± 0.51a	0.96 ± 0.29a	0.65 ± 0.07a
Cacat	0.85 ± 0.11c	0.73 ± 0.06c	1.16 ± 0.04b	1.58 ± 0.06a	0.88 ± 0.07c	0.84 ± 0.05c
Total Flavan−3‐ols	20.03 ± 1.58b	11.44 ± 0.82f	17.50 ± 0.19c	23.93 ± 0.60a	16.05 ± 0.52b	17.47 ± 1.22e
Az	1.03 ± 0.10a	1.00 ± 0.13a	0.58 ± 0.05b	0.91 ± 0.04a	1.01 ± 0.06a	0.86 ± 0.10a
Quara	1.19 ± 0.15a	0.80 ± 0.12bc	0.54 ± 0.03c	0.73 ± 0.00bc	0.82 ± 0.13abc	1.05 ± 0.16ab
Quxyl	1.18 ± 0.10a	0.69 ± 0.13b	0.33 ± 0.01c	0.63 ± 0.02bc	0.68 ± 0.04b	0.81 ± 0.17b
Av	1.22 ± 0.14a	0.69 ± 0.16b	0.31 ± 0.02c	0.69 ± 0.02b	0.73 ± 0.04b	0.88 ± 0.15b
Ka	1.06 ± 0.05a	0.88 ± 0.12ab	0.59 ± 0.10b	0.82 ± 0.04ab	0.75 ± 0.06ab	0.89 ± 0.16ab
Kaglu	1.17 ± 0.12a	0.91 ± 0.15ab	0.53 ± 0.04c	0.82 ± 0.02bc	0.80 ± 0.06bc	0.91 ± 0.15ab
Kagal	1.07 ± 0.08a	0.68 ± 0.17bc	0.39 ± 0.03c	0.74 ± 0.08abc	0.71 ± 0.07bc	0.76 ± 0.15ab
Myara	0.98 ± 0.02a	0.79 ± 0.02b	0.84 ± 0.06ab	0.89 ± 0.06ab	0.87 ± 0.02ab	0.90 ± 0.09ab
Myxyl	0.88 ± 0.08a	0.70 ± 0.02b	0.62 ± 0.04b	0.63 ± 0.02b	0.69 ± 0.03b	0.76 ± 0.08ab
iQuglu	0.99 ± 0.05a	0.99 ± 0.05a	0.82 ± 0.06b	0.95 ± 0.03ab	0.94 ± 0.05ab	0.96 ± 0.03ab
Qugluco	1.00 ± 0.00a	0.91 ± 0.06a	0.79 ± 0.05a	0.91 ± 0.03a	0.97 ± 0.06a	1.02 ± 0.14a
Qugal	0.93 ± 0.04ab	0.88 ± 0.06ab	0.75 ± 0.03b	0.87 ± 0.06ab	1.03 ± 0.02a	0.98 ± 0.10a
Hyglu	1.05 ± 1.05a	0.89 ± 0.89ab	0.62 ± 0.62c	0.78 ± 0.78bc	0.85 ± 0.85ab	0.90 ± 0.90ab
Is	1.09 ± 0.04a	0.82 ± 0.12b	0.59 ± 0.01c	0.78 ± 0.01bc	0.84 ± 0.04b	0.88 ± 0.10ab
sQuglu	1.01 ± 0.02a	0.84 ± 0.05ab	0.69 ± 0.02b	0.92 ± 0.05a	0.96 ± 0.03a	1.03 ± 0.13a
Quglu	1.07 ± 0.04a	0.93 ± 0.06ab	0.91 ± 0.05ab	0.89 ± 0.02b	0.99 ± 0.06ab	1.07 ± 0.05a
*Quglu	0.80 ± 0.00b	0.71 ± 0.08bc	0.58 ± 0.01c	0.76 ± 0.04b	0.73 ± 0.03b	1.02 ± 0.03a
Isglu	0.91 ± 0.10a	1.05 ± 0.13a	0.60 ± 0.02b	0.91 ± 0.03a	0.98 ± 0.10a	1.09 ± 0.04a
Tr	0.86 ± 0.11a	1.01 ± 0.12a	0.60 ± 0.02b	0.90 ± 0.01a	0.98 ± 0.08a	1.05 ± 0.02a
Rh	1.02 ± 0.14a	1.15 ± 0.14a	0.64 ± 0.03b	1.00 ± 0.05a	1.09 ± 0.07a	1.19 ± 0.10a
Mygal	0.82 ± 0.05b	0.73 ± 0.06b	0.73 ± 0.03b	0.72 ± 0.02b	0.76 ± 0.01b	0.99 ± 0.02a
Go	0.99 ± 0.09b	1.18 ± 0.12ab	1.14 ± 0.07ab	1.14 ± 0.05ab	1.26 ± 0.05a	1.05 ± 0.07ab
Goglu	0.79 ± 0.04b	0.73 ± 0.09b	0.71 ± 0.02b	0.67 ± 0.03b	0.73 ± 0.03b	1.00 ± 0.03a
Myglu	1.04 ± 0.02a	0.83 ± 0.05b	0.76 ± 0.03b	0.80 ± 0.03b	0.85 ± 0.05b	0.89 ± 0.08b
My	0.96 ± 0.03a	1.02 ± 0.10a	0.90 ± 0.02a	0.97 ± 0.05a	0.99 ± 0.06a	0.93 ± 0.04a
Qumal	1.38 ± 0.21a	0.63 ± 0.15c	1.25 ± 0.20ab	0.60 ± 0.13c	0.77 ± 0.17bc	0.79 ± 0.14bc
rKaglu	1.03 ± 0.02a	0.69 ± 0.02cd	0.77 ± 0.08cd	0.82 ± 0.02bc	0.67 ± 0.02d	0.95 ± 0.06ab
Kaneo	1.09 ± 0.05a	0.84 ± 0.04bc	0.86 ± 0.02bc	0.94 ± 0.02b	0.76 ± 0.03c	1.09 ± 0.07a
*rQuglu	1.08 ± 0.05a	0.84 ± 0.07c	0.64 ± 0.05d	1.05 ± 0.03ab	0.68 ± 0.00d	0.92 ± 0.06bc
Qurob	1.08 ± 0.05a	0.86 ± 0.13abc	0.67 ± 0.05c	0.88 ± 0.05abc	0.73 ± 0.02bc	0.97 ± 0.13ab
Qu	1.07 ± 0.06ab	0.86 ± 0.07cd	0.66 ± 0.05e	1.13 ± 0.04a	0.69 ± 0.02de	0.91 ± 0.09bc
Qurut	1.25 ± 0.13a	1.01 ± 0.16ab	0.61 ± 0.04c	0.78 ± 0.08bc	0.71 ± 0.07bc	0.94 ± 0.16abc
Quneo	1.06 ± 0.04ab	0.91 ± 0.10b	0.68 ± 0.04c	1.14 ± 0.05a	0.72 ± 0.02c	0.94 ± 0.06b
rQuglu	1.01 ± 0.03a	0.80 ± 0.06c	0.62 ± 0.04d	0.96 ± 0.02ab	0.62 ± 0.02d	0.85 ± 0.04bc
Se	0.93 ± 0.04bc	0.92 ± 0.04bc	0.73 ± 0.01d	1.49 ± 0.00a	0.83 ± 0.02cd	0.99 ± 0.06b
Me	0.90 ± 0.07bc	0.89 ± 0.04bc	0.75 ± 0.04c	1.60 ± 0.13a	0.81 ± 0.03c	1.03 ± 0.02b
Qusop	1.23 ± 0.14ab	1.35 ± 0.15a	0.78 ± 0.04c	1.19 ± 0.02ab	0.88 ± 0.01bc	1.16 ± 0.17ab
Hy	1.16 ± 0.13ab	1.13 ± 0.11ab	0.79 ± 0.01b	1.19 ± 0.01a	0.82 ± 0.09ab	1.03 ± 0.20ab
Hydig	1.19 ± 0.25a	1.03 ± 0.23a	0.78 ± 0.02a	1.57 ± 0.02a	0.88 ± 0.13a	1.13 ± 0.32a
Pa	0.92 ± 0.04c	2.60 ± 0.42a	1.16 ± 0.08bc	2.21 ± 0.13a	1.42 ± 0.04bc	1.62 ± 0.25b
gGoglu	0.94 ± 0.03c	1.20 ± 0.02b	1.14 ± 0.01bc	2.25 ± 0.17a	1.30 ± 0.04b	1.27 ± 0.07b
Pagen	0.84 ± 0.08d	1.97 ± 0.26b	1.34 ± 0.04c	2.70 ± 0.17a	1.35 ± 0.04c	1.32 ± 0.04c
Total flavonoids	43.27 ± 1.36a	40.34 ± 2.83b	30.79 ± 0.27d	43.33 ± 0.66a	36.65 ± 1.27c	41.78 ± 3.48ab

Note

Different lowercase letters indicate significant differences between treatments as calculated by Tukey's HSD test (*p* < .05).

Abbreviations: Ca: Catechin*; Pe: 5,7,3',4',5'‐Pentahydroxyflavan (Tricetiflavan); Ep: Epicatechin*; Hydro: 4'‐Hydroxy‐5,7‐dimethoxyflavanone; Medic: 7‐*O*‐Methyleriodictyol; Meepi: 3'‐*O*‐Methyl‐(‐)‐epicatechin; Ga: Gallocatechin*; Epcat: Epigallocatechin*; Epgal: Epicatechingallate*; Cagal: Catechingallate*; Cid: CinchonainId; βCadih: Catechin‐(7,8‐bc)‐4β‐(3,4‐dihydroxyphenyl)‐dihydro‐2‐(3H)‐one; αCadih: Catechin‐(7,8‐bc)‐4α‐(3,4‐dihydroxyphenyl)‐dihydro‐2‐(3H)‐one; Cic: Cinchonain; Epglu: Epicatechinglucoside; Epiga: Epigallocatechin‐3‐gallate; Na: Naringenin‐7‐*O*‐(6''‐malonyl) glucoside; Gagal: Gallocatechin‐(4α→8)‐gallocatechin; Cacat: Catechin‐catechin‐catechin; Az: Azaleatin (5‐*O*‐Methylquercetin); Quara: Quercetin‐3‐*O*‐arabinoside (Guaijaverin); Quxyl: Quercetin‐3‐*O*‐xyloside (Reynoutrin); Av: Avicularin (Quercetin‐3‐*O*‐α‐L‐arabinofuranoside); Ka: Kaempferol‐7‐*O*‐glucoside*; Kaglu: Kaempferol‐3‐*O*‐glucoside (Astragalin)*; Kagal: Kaempferol‐3‐*O*‐galactoside (Trifolin)*; Myara: Myricetin‐3‐*O*‐arabinoside; Myxyl: Myricetin‐3‐*O*‐xyloside; iQuglu: Quercetin‐3‐*O*‐glucoside (Isoquercitrin)*; Qugluco: Quercetin‐7‐*O*‐glucoside*; Qugal: Quercetin‐3‐*O*‐galactoside (Hyperin)*; Hyglu: 6‐Hydroxykaempferol‐7‐*O*‐glucoside; Is: Isohyperoside; sQuglu: Quercetin‐4'‐*O*‐glucoside (Spiraeoside)*; Quglu: Quercetin‐4′‐*O*‐glucuronide*; *Quglu: Quercetin‐5‐*O*‐glucuronide*; Isglu: Isorhamnetin‐7‐*O*‐glucoside (Brassicin); Tr: Tricin‐4'‐methylether‐3'‐*O*‐glucoside; Rh: Rhamnetin‐3‐*O*‐Glucoside; Mygal: Myricetin‐3‐*O‐*galactoside*; Go: Gossypetin‐8‐*O*‐glucoside*; Goglu: Gossypetin‐3‐*O*‐glucoside*; Myglu: Myricetin‐3‐*O*‐glucoside*; My: Myricetin‐3‐*O*‐glucuronide; Qumal: Quercetin‐3‐*O*‐(6''‐malonyl) galactoside*; rKaglu: Kaempferol‐3‐*O*‐glucoside‐7‐*O*‐rhamnoside; Kaneo: Kaempferol‐3‐*O*‐neohesperidoside; *rQuglu: Quercetin‐3‐*O*‐glucoside‐7‐*O*‐rhamnoside; Qurob: Quercetin‐3‐*O*‐robinobioside; Qu: Quercetin‐7‐*O*‐rutinoside; Qurut: Quercetin‐3‐*O*‐rutinoside (Rutin); Quneo: Quercetin‐3‐*O*‐neohesperidoside; rQuglu: Quercetin‐3‐*O*‐(4''‐*O*‐glucosyl) rhamnoside; Se: Sexangularetin‐3‐*O*‐glucoside‐7‐*O*‐rhamnoside; Me: 6‐C‐Methylquercetin‐3‐*O*‐rutinoside; Qusop: Quercetin‐3‐*O*‐sophoroside (Baimaside); Hy: 6‐Hydroxykaempferol‐3,6‐*O*‐Diglucoside; Hydig: 6‐Hydroxykaempferol‐7,6‐*O*‐Diglucoside; Pa: Patuletin‐3‐*O*‐rutinoside; gGoglu: Gossypetin‐3‐*O*‐glucuronide‐8‐*O*‐glucoside; and Pagen: Patuletin‐7‐*O*‐gentiobioside.

Table 4 shows the main flavonols are quercetin and its derivatives, followed by myricetin, kaempferol, and its derivatives, which account for 57.83% of the total flavonols. From the perspective of individual flavonol types, the contents of Pa, gGoglu, and Pagen were increased by different nitrogen fertilizer types. Pagen, Qusop, and My were the highest under AS treatment, and Qugluco, sQuglu, *Quglu, Isglu, Tr, and Rh were the highest under Glu treatment. Compared with control, Glu treatment significantly upregulated 15 flavonol monomers. In addition to Ur treatment, the total flavonol content of grape peel under other nitrogen fertilizer treatments decreased to different degrees compared with the control, and the content of GAN treatment was the lowest, which decreased by 28.84% compared with the control.

### 3.4 Principal component analysis of flavonoids under nitrogen regulation at veraison stage

**Figure 1:**
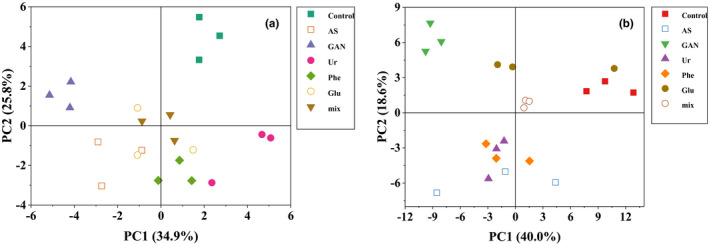
Principal component analysis of flavonoids in grape berries regulated by different nitrogen. Note: (a): Relative content of anthocyanins in grape berries treated with different nitrogen sources; (b): relative contents of flavanols and flavonols in grape berries treated with different nitrogen sources

Principal component analysis (PCA) was used to represent the differences between different treatments (Figure 1). It can be seen from the mix samples in the two figures that the instrument was relatively stable in the detection process, indicating that the data could be used for the following analysis. As can be seen from Figure 1a, for different nitrogen source treatments, PC1 explained 34.9% of the variance, and PC2 explained 25.8% of the variance, accounting for 60.7% of all the variances. In terms of the first principal component, GAN group and Ur group can be well distinguished from other groups, while the metabolite accumulation in AS, Phe, and Glu groups is similar, and the control group can be distinguished from other groups in terms of the second principal component. As shown in Figure 1b, for different nitrogen source treatments, PC1 explained 40.0% of the variance, and PC2 explained 18.6% of the variance, accounting for 58.6% of all the variances. In terms of the first principal component, the GAN group and the control group can be well distinguished from other groups, while the accumulation of metabolites in the GAN, Ur, Phe, and Glu groups is relatively similar, and the Glu group can be distinguished from other groups in terms of the second principal component.

## 4 DISCUSSION

### 4.1 Effects of nitrogen regulation on grape and berry components during veraison stage

Nitrogen can promote the nutritional growth of wine grapes and then affect the quality of wine grapes (Carina et  al., 2019). In this study, it was found that nitrogen regulation at the veraison stage could improve the contents of soluble solids, titratable acids, tannins, anthocyanins, and total acids in grape berries, which was consistent with the conclusion of Cheng et  al., (2020) that proper application of nitrogen fertilizer could improve grapefruit quality and thus improve the contents of various indexes in fruits. The soluble solids of grape berries are the highest in the treatment of ammonium sulfate, and there is no significant difference in the treatment of glutamic acid compared with the control, which is similar to the research results of Janjanin et  al., (2016). Javier et  al., (2017) found that the titratable acid content of grape berries was 5.41% higher than that of the control after applying urea. Similar conclusions were also drawn in this experiment. Nitrogen regulation at the veraison stage had no significant difference in reducing sugar content of grape berries, which was similar to the study of Perez‐Alvarez et  al., (2017). They concluded that leaf nitrogen application had no significant effect on some physicochemical parameters of grape.

### 4.2 Effects of nitrogen regulation on anthocyanins in grape skins during veraison stage

Anthocyanin is a natural colorant existing in the skins of red grapes, which is the fundamental cause of the red appearance of grapes. The proportion and amount of each anthocyanin are greatly affected by varieties and cultivation conditions (Mattivi et  al., 2006; Stéphane et  al., 2004). In this experiment, it was found that nitrogen regulation at the veraison stage had no significant effect on the content of single anthocyanins such as Ptacet and Mvacet, and some studies also showed that leaf nitrogen application rate had almost no effect on the content of anthocyanins in grape (Gutiérrez‐Gamboa et  al., 2017). Urea treatment significantly increased the monomer contents of Cygluc and Dpacet in anthocyanins. Except for Cy, Pt, and cMvcoum, the contents of almost all individual anthocyanins under urea treatment were significantly higher than those under other treatments. Moreover, the total anthocyanin content in grape skins under urea treatment was the highest, which was similar to Javier et  al., (2017). However, this result is in contrast with the research results of Chassy et  al., (2014). Due to different vineyard environmental factors and cultivation management measures, nitrogen types also have different effects on anthocyanins, so the effect of phenylalanine is sometimes better than that of urea (Javier et  al., 2017; Riccardo et  al., 2013).

### 4.3 Effects of nitrogen regulation on flavanols and flavonols in grape skins during veraison stage

Flavanols and flavonols are subgroups of flavonoids and are synthesized mainly in grape skins (González‐Manzano et  al., 2019). In this study, flavanols were mainly catechins, followed by epicatechins, which was also observed by Sergio et  al., (2007). Nitrogen regulation at the veraison stage would increase the contents of Meepi, Epgal, and Cagal, but the contents of other flavanols did not significantly increase compared with the control. Schreiner et  al., (2014) also found that the supply of different kinds of nitrogen fertilizer did not change the contents of catechin, epicatechin, or epicatechin‐3‐gallate. Only the content of total flavanols in low‐concentration urea treatment was significantly higher than that in the control group (Javier et  al., 2015), and this study also concluded that urea treatment could improve the content of total flavanols in grape skins. In summary, urea can increase the total content of flavanols and then improve the quality and taste of wine and grapes.

Flavonols are important pigments that help to stabilize red anthocyanins (Boulton, 2001). Javier et  al., (2015) found that the increase in flavonol content in grapes may improve the quality of wine, because flavonol, as an adjuvant, indirectly affects the formation of wine color. In addition, Ritchey and Waterhouse (1999) found that high‐quality wines contain higher levels of flavonol compounds, indicating that the flavonol content in grapes affects the quality of wine. Javier et  al., (2017) believed that myricetin was the main compound of flavonol, followed by quercetin. Due to the influence of nitrogen application rate, grape growing soil, and climate conditions, different metabolites will be generated. In this experiment, it was found that quercetin and its derivatives are the main flavonols, followed by myricetin, kaempferol, and its derivatives, which is contrary to previous conclusions. Javier et  al., (2017) showed that there was no significant difference in flavonol content in grape skins between control treatment and foliar application of nitrogen fertilizer. In this study, it was also found that except urea, other nitrogen did not increase the flavonol content, or even decreased the flavonol content.

## 5 CONCLUSION

In this study, we determined the effects of nitrogen regulation at the veraison stage on the composition of grape berries and the content of flavonoids in grape skins. The contents of soluble solids, anthocyanins, and total phenols in grape berries were increased by spraying ammonium sulfate on leaf surface. Spraying phenylalanine could increase the content of titratable acid and tannin and decrease the ratio of sugar to acid. Compared with the control, foliar spraying of nitrogen fertilizer will increase the content of some flavonoid monomers. And the treatment of spraying urea can significantly increase the content of total anthocyanins, total flavanols, and total flavonols in wine grape skins. These results have important oenological significance for grape quality.

## CONFLICT OF INTEREST

The authors declare that they have no competing interests.

## AUTHOR CONTRIBUTION

**Yueran Hui:** Conceptualization (lead); Data curation (equal); Formal analysis (equal); Software (lead); Writing‐original draft (equal). **Jing Wang:** Data curation (equal); Formal analysis (equal); Investigation (lead); Writing‐original draft (equal). **Tingting Jiang:** Writing‐original draft (equal); Writing‐review & editing (equal). **Tinghui Ma:** Data curation (equal); Writing‐original draft (equal). **Rui Wang:** Funding acquisition (lead); Writing‐review & editing (lead).

## Data Availability

The data that support the findings of this study are available from the corresponding author upon reasonable request.

## References

[fsn32503-bib-0001] Bell, S. J. , & Henschke, P. A. (2005). Implications of nitrogen nutrition for grapes, fermentation and wine. Australian Journal of Grape & Wine Research, 11(3), 242–295. 10.1111/j.1755-0238.2005.tb00028.x

[fsn32503-bib-0002] Bin, T. , Roland, H. , James, M. , & Marlene, J. (2019). Changes in pathogenesis‐related proteins and phenolics in Vitis vinifera L. cv. ‘Sauvignon Blanc’ grape skin and pulp during ripening. Scientia Horticulturae, 243, 78–83. 10.1016/j.scienta.2018.08.018

[fsn32503-bib-0003] Boulton, R. (2001). The copigmentation of anthocyanins and its role in the color of red wine: A critical review. American Journal of Enology & Viticulture, 52(2), 67–87.

[fsn32503-bib-0004] Carina, P. L. , Nikolaus, M. , Iris, K. , Jens, P. , & Christian, Z. (2019). Different forms of nitrogen application affect metabolite patterns in grapevine leaves and the sensory of wine. Plant Physiology and Biochemistry, 143, 308–319. 10.1016/j.plaphy.2019.09.009 31539760

[fsn32503-bib-0005] Chassy, A. W. , Adams, D. O. , & Waterhouse, A. L. (2014). Tracing phenolic metabolism in Vitis vinifera berries with 13C6‐phenylalanine: Implication of an unidentified intermediate reservoir. Journal of Agricultural and Food Chemistry, 62(11), 2321–2326. 24611798 10.1021/jf402229u

[fsn32503-bib-0006] Chen, Y. X. , Jia, Y. , Cui, N. B. , Yang, Y. G. , Zhao, L. , Hu, X. T. , & Gong, D. Z. (2018). Effects of integrated management of water and fertilizer on citrus photosynthesis, yield and water use Efficiency. Journal of Irrigation and Drainage, 37(S2), 50–58.

[fsn32503-bib-0007] Cheng, X. H. , Ma, T. T. , Wang, P. P. , Liang, Y. Y. , Zhang, J. X. , Zhang, A. , Chen, Q. Y. , Li, W. P. , Ge, Q. , Sun, X. Y. , & Fang, Y. L. (2020). Foliar nitrogen application from veraison to preharvest improved flavonoids, fatty acids and aliphatic volatiles composition in grapes and wines. Food Research International, 137, 109566. 10.1016/j.foodres.2020.109566 33233183

[fsn32503-bib-0008] Garde‐Cerdán, T. , & Ancín‐Azpilicueta, C. (2008). Effect of the addition of different quantities of amino acids to nitrogen‐deficient must on the formation of esters, alcohols, and acids during wine alcoholic fermentation. LWT‐Food Science and Technology, 41(3), 501–510. 10.1016/j.lwt.2007.03.018

[fsn32503-bib-0009] Garde‐Cerdán, T. , Santamaría, P. , Rubio‐Bretón, P. , González‐Arenzana, L. , López‐Alfaro, I. , & López, R. (2015). Foliar application of proline, phenylalanine, and urea to tempranillo vines: Effect on grape volatile composition and comparison with the use of commercial nitrogen fertilizers. LWT ‐ Food Science and Technology, 60(2), 684–689. 10.1016/j.lwt.2014.10.028

[fsn32503-bib-0010] González‐Manzano, S. , Montserrat, D. , Rivas‐Gonzalo, J. C. , Escribano‐Bailón, M. T. , & Santos‐Buelga, C. (2019). Studies on the copigmentation between anthocyanins and flavan‐3‐ols and their influence in the colour expression of red wine. Food Chemistry, 114(2), 649–656. 10.1016/j.foodchem.2008.10.002

[fsn32503-bib-0011] Gonzalo, G. B. , Astrid, B. , Julia, S. , Anscha, J. J. Z. , William, G. T. W. , John, P. M. , & Wessel, J. D. T. (2019). Investigating the relationship between grape cell wall polysaccharide composition and the extractability of phenolic compounds into shiraz wines. Part II: Extractability during fermentation into wines made from grapes of different ripeness levels. Food Chemistry, 278, 26–35. 30583371 10.1016/j.foodchem.2018.10.136

[fsn32503-bib-0012] Gutiérrez‐Gamboa, G. , Garde‐Cerdán, T. , Javier, P. , & Martínez‐Gil, A. M. (2017). Foliar nitrogen application in Cabernet Sauvignon vines: Effects on wine flavonoid and amino acid content, Food Research International, 96, 46‐53. 28528107 10.1016/j.foodres.2017.03.025

[fsn32503-bib-0013] Hannam, K. D. , Neilsen, G. H. , Neilsen, D. , Midwood, A. J. , Millard, P. , Zhang, Z. I. , & Steinke, D. (2016). Amino acid composition of grape (Vitis vinifera L.) juice in response to applications of urea to the soil or foliage. American Journal of Enology & Viticulture, 67, 47–55.

[fsn32503-bib-0014] Janjanin, D. , Karoglan, M. , Ćustić, M. H. , Bubola, M. , Osrečak, M. , & Palčić, I. (2016). Response of ‘Italian Riesling’ leaf nitrogen status and fruit composition (Vitis vinifera L.) to foliar nitrogen fertilization. HortScience, 51, 262–267. 10.21273/HORTSCI.51.3.262

[fsn32503-bib-0015] Javier, P. , López‐Alfaro, I. , Gómez‐Alonso, S. , López, R. , & Garde‐Cerdán, T. (2015). Changes on grape phenolic composition induced by grapevine foliar applications of phenylalanine and urea. Food Chemistry, 180, 171–180. 10.1016/j.foodchem.2015.02.042 25766815

[fsn32503-bib-0016] Javier, P. , Pilar, S. , Rosa, L. , & Garde‐Cerdán, T. (2017). Phenolic composition of Tempranillo grapes following foliar applications of phenylalanine and urea: A two‐year study. Scientia Horticulturae, 219, 191–199. 10.1016/j.scienta.2017.03.014

[fsn32503-bib-0017] Jin, Z. X. , Sun, H. , Sun, T. Y. , & Yao, Y. X. (2016). Modifications of 'gold finger' grape berry quality as affected by the different rootstocks. Journal of Agricultural and Food Chemistry, 64(21), 4189–4197. 10.1021/acs.jafc.6b00361 27088562

[fsn32503-bib-0018] Liang, N. N. , Pan, Q. H. , He, F. , Wang, J. , Reeves, M. J. , & Duan, C. Q. (2013). Phenolic profiles of Vitis davidii and Vitis quinquangularis species Native to China. Journal of Agricultural and Food Chemistry, 61(25), 6016–6027. 10.1021/jf3052658 23721215

[fsn32503-bib-0019] Ma, T. T. , Wang, J. Q. , Wang, H. L. , & Sun, X. Y. (2020). Is overnight fresh juice drinkable? The shelf life prediction of non‐industrial fresh watermelon juice based on the nutritional quality, microbial safety quality, and sensory quality. Food & Nutrition Research, 24. 10.29219/fnr.v64.4327 PMC7307432 32612491

[fsn32503-bib-0020] Martínez‐Lüscher, J. , Chen, L. C. C. , Brillante, L. , & Kurtural, S. K. (2017). Partial solar radiation exclusion with color shade nets reduces the degradation of organic acids and flavonoids of grape berry (Vitis vinifera L.). Journal of Agricultural and Food Chemistry, 65(49), 10693–10702. 29141407 10.1021/acs.jafc.7b04163

[fsn32503-bib-0021] Mattivi, F. , Guzzon, R. , Vrhovsek, U. , Stefanini, M. , & Velasco, R. (2006). Metabolite profiling of grape: Flavonols and anthocyanins. Journal of Agricultural and Food Chemistry, 54(20), 7692–7702. 17002441 10.1021/jf061538c

[fsn32503-bib-0022] Miquel, P. , Josep, M. V. , & Josep, R. (2016). Water use efficiency in peach trees over a four‐years experiment on the effects of irrigation and nitrogen application. Agricultural Water Management, 164, 253–266. 10.1016/j.agwat.2015.10.021

[fsn32503-bib-0023] Perez‐Alvarez, E. P. , Garde‐Cerdán, T. , García‐Escudero, E. , & Martínez‐Vidaurre, J. M. (2017). Effect of two doses of urea foliar application on leaves and grape nitrogen composition during two vintages. Journal of the Science of Food and Agriculture, 97, 2524–2532. 10.1002/jsfa.8069 27704545

[fsn32503-bib-0024] Riccardo, F. , Fulvio, M. , Mirko, R. , Panagiotis, A. , & Luigi, B. (2013). Advanced knowledge of three important classes of grape phenolics: Anthocyanins, stilbenes andflavonols. International Journal of Molecular Sciences, 14(10), 19651–19669. 10.3390/ijms141019651 24084717 PMC3821578

[fsn32503-bib-0025] Ritchey, J. G. , & Waterhouse, A. L. (1999). A standard red wine: Monomeric phenolic analysis of commercial Cabernet Sauvignon wines. American Journal of Enology and Viticulture, 50(2), 91–100.

[fsn32503-bib-0026] Schreiner, R. P. , Scagel, C. F. , & Lee, J. (2014). N, P, and K supply to pinot noir grapevines: Impact on berry phenolics and free amino acids. American Journal of Enology & Viticulture, 65(1), 43–49. 10.5344/ajev.2013.13037

[fsn32503-bib-0027] Sergio, G. A. , Esteban, G. R. , & Isidro, H. G. (2007). HPLC analysis of diverse grape and wine phenolics using direct injection and multidetection by DAD and fluorescence. Journal of Food Composition and Analysis, 20(7), 618–626. 10.1016/j.jfca.2007.03.002

[fsn32503-bib-0028] Sohrab, D. , Ali, T. , Gholamhossein, D. , Javier, A. , & Reza, K. (2016). Effects of foliar applications of zinc and boron nano‐fertilizers on pomegranate (Punica granatum cv. Ardestani) fruit yield and quality. Scientia Horticulturae, 210, 57–64. 10.1016/j.scienta.2016.07.003

[fsn32503-bib-0029] Stéphane, V. , Leigh, F. , Ann, N. , Mariola, K. , Véronique, C. , & Elizabeth, W. (2004). Taste and mouth‐feel properties of different types of tannin‐like polyphenolic compounds and anthocyanins in wine. Analytica Chimica Acta, 513(1), 57–65. 10.1016/j.aca.2003.10.017

[fsn32503-bib-0030] Wei, X. F. , Ju, Y. L. , Ma, T. T. , Zhang, J. X. , Fang, Y. L. , & Sun, X. Y. (2020). New perspectives on the biosynthesis, transportation, astringency perception and detection methods of grape proanthocyanidins. Critical Reviews in Food Science and Nutrition, 3, 1–27. 10.1080/10408398.2020.1777527 32551848

[fsn32503-bib-0031] Yang, H. (2016). Research on the quality and wine‐making characteristics of the main grape varieties grown in different regions of Jilin Province. Changchun: Jilin Agricultural University.

[fsn32503-bib-0032] Zhang, G. J. , Wang, X. Y. , Sun, L. , Yan, A. L. , Ren, J. C. , Wang, H. L. , & Xu, H. Y. (2017). Growing technique of two seasons a year for greenhouse grapes in Beijing area. Chinese and Foreign Grapes and Wine, 5, 49–55.

[fsn32503-bib-0033] Zhang, L. X. , Zhang, L. S. , Li, B. Z. , & Han, M. Y. (2007). Mineral nutrition elements and their roles in growth and development of apple trees in arid areas. Journal of Northwest Forestry University, 3, 111–115.

[fsn32503-bib-0034] Zhao, C. X. , Yu, J. H. , Feng, Z. , He, Z. X. , Lu, J. Z. , Yu, J. , Dong, A. L. , Wang, L. M. , & Pu, R. F. (2017). Effects of controlled‐release fertilizers on yield, quality and nutrient use efficiency of tomato under substrate culture. Journal of Gansu Agricultural University, 52(2), 34–40.

[fsn32503-bib-0035] Zhao, Y. , Liu, H. F. , Zhao, Q. X. , Zheng, X. N. , Niu, Y. , & Ma, F. Y. (2018). Effects of different irrigation rates in facility conditions on photosynthetic physiological characteristics and quality formation during ripening period of Frey Seedless Grape. Xinjiang Agricultural Reclamation Science and Technology, 41(5), 11–15.

